# The Introduction of Medication-Free Mental Health Services in Norway: An Analysis of the Framing and Impact of Arguments From Different Standpoints

**DOI:** 10.3389/fpsyt.2021.685024

**Published:** 2021-07-19

**Authors:** Olav Nyttingnes, Jorun Rugkåsa

**Affiliations:** ^1^Health Services Research Unit, Akershus University Hospital, Lørenskog, Norway; ^2^R&D Department Mental Health, Akershus University Hospital, Lørenskog, Norway; ^3^Centre for Care Research, University of South-Eastern Norway, Porsgrunn, Norway

**Keywords:** medication-free treatment, coercion, mental health care, antipsychotics, user organizations, mental health discourses, experiential knowledge, discourse analysis

## Abstract

**Introduction:** Debates about coercive practices have challenged a traditional biomedical hegemony in mental health care. The perspectives of service user organizations have gained considerable ground, such as in the development of the Convention on the Rights of Persons with Disabilities. Such changes are often contested, and might in practice be a result of (implicit) negotiation between stakeholders with different discursive positions. To improve understanding of such processes, and how discursive positions may manifest and interact, we analyzed texts published over a 10 year period related to the introduction of medication-free inpatient services in Norway.

**Methods:** We conducted qualitative analyses of 36 policy documents related to the introduction of medication-free services and 75 opinion pieces from a subsequent debate. We examined discursive practices in these texts as expressions of what is perceived as legitimate knowledge upon which to base mental health care from the standpoints of government, user organizations and representatives of the psychiatric profession. We paid particular attention to how standpoints were framed in different discourse surrounding mental health care, and how these interacted and changed during the study period (2008–2018).

**Results:** The analysis shows how elements from the discourse promoted by service user organizations—most notably the legitimacy of personal experiences as a legitimate source of knowledge—entered the mainstream by being incorporated into public policy. Strong reactions to this shift, firmly based in biomedical discourse, endorsed evidence-based medicine as the authoritative source of knowledge to ensure quality care, although accepting patient involvement. Involuntary medication, and how best to help those with non-response to antipsychotic medication represented a point at which discursive positions seemed irreconcilable.

**Conclusion:** The relative authorities of different sources of knowledge remain an area of contention, and especially in determining how best to help patients who do not benefit from antipsychotics. Future non-inferiority trials of medication-free services may go some way to break this discursive deadlock.

## Introduction

Coercion, in the form of involuntary care, seclusion, restraints or involuntary medication is a controversial aspect of psychiatric practice. Several countries, including Norway, express policy ambitions to reduce the use of coercion (1, 2). Many patient activists^1^ and their advocates have long challenged coercive practices and lobbied for the protection from involuntary care through autonomy-based approaches (4) and a focus on recovery (5). Broadly speaking, the last 60 years have seen continuous efforts from user organizations and their academic, clinical, legal or political allies to challenge the traditional hegemony in psychiatric services through political or legal processes (6). These efforts have had some effects. For example, in the development of the Convention on the Rights of Persons with Disabilities (CRPD) (7, 8) user organizations were instrumental in replacing a “medical” model of disability and mental disorders with a “social” one, and setting out a drastically limited scope for involuntary care (9). Implementation of the Convention is slow to materialize (10) despite ratification by the governments in 181 countries. A number of academics and practitioners from the fields of psychiatry and law argue that in banning all guardianship and coerced treatment, the CRPD does not strike the right balance between patient autonomy and the professional duty to protect patients (11, 12). Patient autonomy was also a decisive factor when involuntary medication was (temporarily) considered unlawful by the German Federal Supreme Court in 2011; this too happened against the opinion expressed by professional associations (13).

In this article, we examine the introduction of medication-free inpatient services (MFS) into national policy for reducing coercion in Norway (14). The process, which eventually took 8 years, was set in motion after being suggested by a service user organization arguing for patients' rights not to be coercively treated with antipsychotic medication. The example of MFS is interesting because antipsychotic medication (under coercion if deemed necessary) remains central to clinical guidelines for both inpatient and outpatient psychosis treatment (15, 16), but remains a core area of conflict (17), and is repeatedly pointed out by patients who have experienced it as the most problematic aspect of coercion (18, 19). Also, once the Norwegian government made MFS mandatory and implementation started, a heated public debate began in which arguments for and against MFS were rehearsed. A close examination of how various stakeholders argued and lobbied for their standpoints in the implementation process and the debate that followed, might shed light on the dynamics of how positions develop and interact over time as regards coercion in mental health care, and what potential sticking points might be. In doing so, we draw particular attention to discursive positions and acts.

### Discursive Positions in the Field of Psychiatry

A *discourse* can be described as “a particular way of talking about and understanding the world (or an aspect of the world)” (20). It implies ways of framing or talking about a subject, it promotes certain mindsets and actions, and can illuminate what a particular actor sees as legitimate knowledge or moral conduct (21). A myriad discourses can be invoked by or observed in expressions about mental health care, be it from patient, carer or clinicians' perspectives or social, historical or popular science spheres. There is no clear consensus about what constitutes and characterizes the main discourses surrounding psychiatry and mental suffering, but a number of perspectives are of relevance for the empirical analysis we present in this article.

*Biomedical discourse* is usually portrayed as understanding mental disorders as illnesses of the brain that require input from psychiatrists (22), who possess the relevant knowledge and therefore the legitimate authority to diagnose and treat (23). Involuntary care is sometimes needed to compensate for patients' lack of insight, in order to ensure that they benefit from evidence-based medicine (EBM) (24). Biomedical discourse is rooted in 19th-century understandings of “madness” (25), and attention was later directed toward impact on mental disorders from psychological and social factors, but medication has remained the central form of treatment (26). This discourse became hegemonic and influenced the development of mental health legislation and the institutionalization of mental health care (23).

In the wake of World War II, new forms of treatment for mental illness were developed in the fields of psychology, nursing, and social work, such as behavior therapy (27) and “the therapeutic community” (28). This was promoted by *psy discourse*, emphasizing that mental distress can be alleviated by changing someone's beliefs, behaviors, or social milieu. This paved the way for multidisciplinary approaches in both inpatient and outpatient settings. Psy discourse criticized the dominant focus on medication and facilitated a division of labor between mental health professionals, promoting a more diverse set of legitimate sources of knowledge and wider approaches to treatment (29). The bio-psycho-social model (30) can be seen as a framework combining biomedical and psy discourse.

It is common to label a number of discursive positions critical of psychiatric practice under the umbrella term *antipsychiatry*. The term was first used in 1908 by a German psychiatrist to describe the oppositional user-movement in Germany at the time (31). The term was reintroduced by David Cooper in the 60 s, and has since often been used to describe the diverse, and in part contradictory, perspectives of Laing, Foucault, Goffman, and Szasz. While none of these figures applied the term to their own scholarship (32), it has become associated with their ideas. Laingian ideas of “madness” as a reasonable response to detrimental circumstances, and the need to meaningfully engage with deeply disturbed patients (32) who are possibly made worse by asylum treatment (33), Foucault's ideas of a great “disciplining” confinement, and Szasz' claim that mental disorders are not real illnesses, are all associated with *antipsychiatry* (32). The same is Goffman's critique of the depersonalization that occur in “total institutions” (32) and the related attempts by Basaglia to replace such institutions with “democratic psychiatry” (34). Common for these approaches is an orientation toward social science, and in particular phenomenology, as sources of knowledge by which to understand psychiatric practice. While some service user organizations embrace the term of antipsychiatry, it is often applied by others as a derogatory term to describe, silence or ignore the potential merit of critical positions (33).

A less critical perspective that nonetheless evolved in opposition to what was perceived as therapeutic pessimism toward those with psychotic disorders is expressed in discourse of *recovery*. Emphasis is placed on how patients often do recover (35) and that this is achieved in a variety ways, and may well happen after someone abandons standard psychiatric treatment (36, 37). Recovery is portrayed as a fundamentally personal process (38), and directs attention toward the individual's hope, meaning, recognition and acceptance (6), which helps them to actively change attitudes and behavior (39). Over the last 20 years, recovery has become central as a guiding principle for the development of mental health services (40). The recovery concept has been criticized for lacking clarity (41), for promoting an individualistic approach, and that the focus on individuals' strengths implicitly mirrors their perceived weaknesses or deficits (42, 43).

Other positions critical of psychiatric practice direct focus toward the structural dimensions shaping it. *Social justice discourse* is concerned with the just distribution of benefits and burdens, the fairness of policy and the access to, and outcome of, public services. This is related to the field of psychiatry in different ways including how poverty is a determinant of mental ill health (44, 45), the curtailment of individual rights to autonomy (46, 47), and the quality of clinical interactions, especially those experienced as degrading and humiliating (4, 48, 49). A concern with distributive, procedural and interactional justice draw both on philosophical inquiry quoting a diverse field of philosophers including Plato, Kant, Mill and Rawls (50) as well as the personal accounts of patients.

Another common source of criticism toward psychiatric practice is that which primarily is concerned with how medicine and psychiatry has allowed the pharmacological industry to gain undue influence in research (51), diagnostic systems (52) and clinical practice (53). Such *pharmaceutic-critical discourse* is often founded on the re-interpretation of pharmaceutical studies, analyses of undesirable interactions, and often view industry-sponsored studies with suspicion (54, 55).

As already alluded to, the emphasis on patient experiences is part of several discursive positions. Over the recent decades, a more explicit *experiential discourse* has evolved that centers on patients' personal experiences of mental disorders and treatment as an authoritative source of knowledge (56). It developed through services users forming alliances, which has gradually increased the influence of this form of knowledge, partly borrowing from the consumerist movement (6). It is often combined with other types of criticisms of current mental health practice, including discourse associated with antipsychiatry, recovery or pharmaceutic-critical discourse.

As a slightly different kind of discursive position, but one of importance for the analysis that follows, is a *bureaucratic discourse*, which views the government as holding legitimate authority to steer and control those acting on behalf of the state, including the mental health professions. This discourse is manifest in arguments and arrangements that define the scope and monitor the conduct of professional powers. Governments' strategies to direct, regulate, change and monitor mental health care can be seen as a attempts at controlling and containing a powerful profession (57). In determining the boundaries of mental health services, is not uncommon that Governments, when expressing their justification for permitting coercive practices, draw on discourse surrounding the assumed *dangerousness* of those with mental illness thus associating mental disorders with criminality and violence (58) often triggered by high profile cases or vivid media depictions (58–60).

Any particular discourse (including those just described) seldom manifests alone or in its purest form. In texts about modern mental health care, different perspectives are usually intertwined and combined, such as when services are described as recovery-oriented and centered around patients' experiences, with an aim to change their circumstances through multidisciplinary efforts, and also sometimes insist on medication (61). While a particular discourse may be associated with one stakeholder group, there is often internal disagreement, conflict, and debates within such groups (22). Discourses are not stable but might change by such internal debate, by incorporating elements from other discourses or through mutual struggle for hegemony. The relative influence of different discursive positions on policy and practice therefore also changes over time (62).

### The Aim of the Article

Discursive framing of social issues can both reflect and contribute to social and cultural change, and an analysis of the interaction between different positions may further understanding of such change (20). By identifying discursive practices, actions and reactions in texts related to the introduction of MFS in Norway, we examine these dimensions in how MFS emerged and relates to coercion. Specifically, we seek to answer three questions:

- How did the policy decision to make MFS mandatory evolve, and which positions and shifts were observed in the process?
- What were the central themes and areas of contention in the public debate that followed the introduction of MFS?
- Based on the standpoints expressed by different stakeholder groups, how can we conceptualize the apparent incommensurability of their positions?

## Materials and Methods

Norway has extensive public health and welfare services. There is tradition for local variation in the development and delivery of public services, but specialist mental health care is ultimately the responsibility of the Ministry of Health, which instructs four Regional Health Authorities through annual Commissioning Letters. The Regional Authorities are responsible for a total of 20 local Hospital Trusts, which design and deliver inpatient and outpatient services. In 2016 there were 86 beds (63) and 38 700 outpatient consultations (64) per 100 000 adult population. In 2017 there were 179 involuntary admissions per 100 000 adult population (65), which in an international context is relatively high (66).

### Data

Written documents can be understood as attempts to commit to paper one's position and justifying it to others. In policy processes, documents are commonly used to promote, impact and influence, and to highlight some issues and downplay or hide others (67). We considered documents concerning MFS as an appropriate source of data for our purpose.

To answer the first research question, we collected all publicly available policy documents that contained information about or views on the introduction of MFS between 2008 (the first identified document) and 2016. A number of such documents were issued by organizations or bodies like the Ministry of Health, Regional Health Authorities, Hospital Trust, and user organizations. Because policy documents are usually published on the internet, we conduced comprehensive online searches using a range of Norwegian terms applicable to MFS. We also conduced targeted searches on the websites of relevant bodies. Through this we identified a total of 36 policy documents. We consulted key individuals in the above organizations to identify any additional documents: two more documents were identified and included. A total of 36 documents thus formed part of the analysis.

We sought to answer our second question using all articles and opinion pieces that constituted the public debate. A total of 75 such texts were identified through comprehensive online searches, and appeared from summer 2016 and the following 2 years, in *Dagens Medisin* (Today's Medicine, a biweekly health sector newspaper, 36 texts), *Aftenposten* (the largest Norwegian subscription newspaper, 16 texts), and *Journal of the Norwegian Medical Association* (15 texts). The remaining texts were published in other newspapers and profession-based journals.

### Analysis

We applied a combination of qualitative analysis methods in three analytical stages, corresponding to our three research questions.

First, we conducted a manifest content analysis (68) of the 36 policy documents to identify their key content and how this was phrased. We looked for connections between texts (20) and how they had impact on each other. This was achieved through producing condensed notes of each text, including any prominent discursive expressions. These notes were used to identify three distinct phases in the development of MFS, which were marked by differences in discursive acts and positioning. This facilitated an examination of the unfolding relationships between and changes in positions.

Second, we conducted an interpretive thematic analysis of all 75 texts from the public debate, in order to arrive at key themes and patterns between them (69). Close reading of all data led to identification of inductive codes, which were refined through an iterative process that paid attention to how various issues in the debate were promoted, addressed, or countered. Since the main stakeholder groups produced opposing texts, we endeavored to read all texts both in an *engaged* way (i.e., seeking to understand the intentions and viewpoints of those expressing a view) and an *estranged* way (i.e., to identify inconsistencies or rhetorical devices) as recommended by Janks (70). We added theoretical codes for the discursive positions identified in stage one. We connected codes into five main analytical themes, which are reported in section The Public Debate.

Third, we used an interpretive approach to arrive at an explanation (70) of why, despite some shifts, observable discursive distance remained between stakeholder groups' expressions. While policy proposals, such as MFS, contain explicit or implicit diagnoses of the “problem” that the policy intervention is intended to solve (38), “problem representations” are not necessarily shared by different stakeholder groups (71). Disagreements are important to detect as they might reflect differing criteria for judging the potential or success of an intervention (71). Using established methods for policy analysis (38, 41), we therefore distilled and critically assessed the “problem representations”(72) of the three main stakeholder groups: the Joint Action for Medication Free Services together with other MFS supporters, the Ministry of Health, and MFS critics. This included reexamining the results from the previous analytical stages, in an iterative interpretive process in which we considered, for each stakeholder groups, their problem representation and how it came about, which premises or assumptions underpinned it, what it left unproblematized, and how it was defended or questioned (62). We paid particular attention to whether stakeholders omitted topics that were central to the arguments of others, as this might in itself constitute a position or point toward conceptual premises underlying a particular standpoint (72). We also revisited the engaged and estranged interpretations of the texts (41).

Below we include excerpts from the texts (translated into English by the authors) to illustrate and validate our interpretations (46). The list of all documents included in the analysis is available on request.

## Results

### How MFS Became a Policy Solution

Key developments in the evolution of MFS as a policy solution, grouped into three phases, are detailed in Table 1.

**Table 1:** Sequence of key arguments and documents^*^ related to the introduction of medication-free services (MFS) in Norway.

	**Date**	**Author**	**Document type**	**Main content related to MFS**
2008-2010: Placing MFS on the policy agenda	May 2008	We Shall Overcome (73)	User organization's presentation to a consultative parliamentary meeting on how to reduce coercion in mental health care	Criticizes current practices and proposes medication-free acute services for those who avoid services due to fears of involuntary medication
	Jun. 2009	The Norwegian Directorate of Health (74)	Task force report discussing the criteria for compulsion and suggesting actions to reduce coercion	Proposes the testing of MFS for inpatient treatment of psychosis as one of 38 suggested actions
	Mar. 2010	Ministry of Health and Care (75)	Amendment to the 2010 Commissioning Letter to the Regional Health Authorities	Announces a forthcoming strategy to implement alternatives to coercion; MFS is one of 11 measures described as the minimal requirements to be planned for
2011-2014: Stakeholders responding and adapting to the Ministrys instriction	2011–2012	Norwegian Health Authorities (76–79)	Four-year plans for reducing coercion	Provide an overview of existing direction and set out frameworks for action plans in local Health Trusts; MFS is not planned for in any Trust
	Oct. 2011	Joint Action (80)	Letter to the Minister of Health	Five NGOs demands actions to ensure that Trusts follow up on the instruction to establish MFS
	Jul. 2012	Ministry of Health and Care (81)	National strategy for reduced coercion and increased voluntariness in mental health services	Sets out the strategy that includes systematic introduction of alternatives to coercion, including MFS
	Oct. 2012	Joint Action (82)	Letter to the Minister of Health	Reiterates that MFS has not been established or planned by any Trust; proposes progress through dialogue with user organizations
	Dec. 2012	Ministry of Health and Care (83)	Commissioning Letter to Regional Health Authorities for 2013	Reiterates that Trusts must implement voluntary services, including MFS
	2012–2014	Various health trusts, including Møre og Romsdal (84), St.Olavs Hospital (85), Stavanger Health trust (86), University hospital of North Norway (87)	Local Trusts' plans for reducing the use of coercion	Describe local plans that vary in form and content regarding which measures to implement to reduce coercion; none include plans to implement MFS
	2013	Joint Action (88)	Positioning document	Summarizes that five national user and carer organizations are united in their demand for patients' right to choose MFS
2015-2016: A bureaucratic assertion of authority	Jan. 2015	Ministry of Health and Care (89)	Commissioning Letter to Regional Health Authorities for 2015	Reiterates the instruction to implement MFS; specifies that these shall be developed in cooperation with user organizations, and sets a reporting deadline
	Nov. 2015	Ministry of Health and Care (90)	Amendment to the 2015 Commissioning Letter to Regional Health Authorities	Firms up the previous instruction with a revised time schedule: five MFS units must be established by June 2016
	2016	Norwegian Regional Health Authorities	Protocols for MFS, reported in Bjørgen et al. (91)	Plans for piloting MFS

* *As there was considerable overlap/duplication in the arguments put forward by stakeholders in the included policy documents, we do not describe all 36 here*.

#### Placing MFS on the Policy Agenda (2008–2010)

The first mention we could find of MFS was in a presentation given by the user organization We Shall Overcome to a consultative parliamentary hearing in 2008, where it was presented as a measure to reduce coercion in acute care. This organization positions itself as critical to psychiatric practice and emphasizes the need to place patient autonomy at the core of mental health care (92). The document criticized the discriminatory nature of mental health legislation on the basis that “*a separate legislation for mental health care reinforces the attitude that those with severe mental disorders are a group of people so different that the Patient Rights Act does not apply to them.”* It was maintained that “*to be coerced to take mediation you do not wish to”* was a breach of the right to autonomy, which “*many experience as harmful, both physically and mentally, amounts to serious abuse* (73). Drawing on social justice discourse and experiential discourse, respectively, these two statements illustrate the document's sharp criticism of the field of psychiatry, also partly resembling some antipsychiatric positions as expressed by Laing and Basaglia. They further argued that MFS would alter the treatment milieu and could contribute toward a recovery-based approach that would align with a patient's preferences: “*For many, who on occasion need support around the clock, the coercion/pressure to take medication represents an obstacle to seeking help. Offering this group treatment without medication could prevent coercion”* (73). The document thus set forth the case for MFS using an amalgamation of discourse: social justice, experiential, antipsychiatry, recovery and psy. Biomedical and pharmaceutic-critical discourse was not used.

The following year, a task force was appointed by the Directorate of Health to assess the criteria for involuntary treatment and evaluate the current action plan for reduced coercion (93). In their report, the task force, in which users, health professionals, researchers, and law experts participated, noted a lack of progress on action to reduce coercion, and listed 38 suggested measures for a possible revised plan. One of these rather vaguely suggested to “*explore whether it would be feasible to test out medication free inpatient treatment as an alternative to traditional psychosis treatment”* (74). No detail as to how this should be done was provided.

Some months later, in March 2010, the Ministry of Health issued an amendment to their annual Commissioning Letter, and with reference to the task force's report, they instructed Health Authorities to bring down the rate of coercion. The rationale included the “*…repeated criticism from users, family carers, and their organizations; claims of rights infringements from UN Human Rights agencies; and [national] statistics…that show that the use of coercion has not decreased…and [that there are] large geographic variations”* (75). The Ministry thus explicitly drew on social justice discourse and the standpoints of user organizations when explaining their position. The letter also pointed to a future strategy for reduced coercion and instructed Trusts to prepare regional and local plans, with a minimum of 11 specified elements, one of which were the “*systematic introduction of alternatives to coercion, including medication-free treatment, patient-controlled admissions, ambulant teams, and individual care plans,”* thus also including psy and recovery perspectives. The Ministry was clear that they expected health professionals to work together with patient representatives to reach these aim, and that the process should reflect the spirit of user involvement and a focus on recovery (75). The instruction was explicit and direct, and the document did not refer to any consultation with psychiatrist organizations, health authorities or trusts, as is common when introducing such changes. That the instruction was issued by the Ministry itself and not one of their executive bodies added to its authority. First proposed by a user organization and only briefly suggested by the Directorate's Task Force, the Ministry now decided to make MFS a requirement and expressed this in language that recognized many of the concerns of the user movement.

#### Stakeholders Responding and Adapting to the Ministry's Instruction (2011–2014)

All four Regional Health Authorities responded by devising plans for reducing the use of coercion (76–79), confer Table 1. While these plans mentioned the requirement of introducing MFS, none of them included any action for doing so. This omission was left unexplained. The lack of progress triggered five national user and carer organizations to form the Joint Action and write to the Health Minister in October 2011. They demanded action and called for “*at least one medication-free acute ward in each Hospital Trust,”* arguing that many patients do not seek help during a crisis because medication—often coerced—was the only treatment offered. What was needed during acute phases of illness, they stated, still drawing on psy and recovery discourse, was “*a safe place to be, a bed to sleep in, regular meals, and people to talk to*.” “Medication-free” should be understood as the absence of coerced medication and treatment pressure, with medication provided “*only when the patient chooses it freely”* (80). This, rather brief, letter was firmly focused on the future, and was not premised on the social justice or antipsychiatric discourse that had been used when first proposing MFS.

The Ministry reiterated the requirement to implement MFS in 2012, this time as part of a national strategy to reduce coercion (81) (a reminder of which was also mentioned in their annual Commissioning letter for 2012) (83). Representatives of health professionals and user organizations had taken part in developing the strategy, and the text expressed the Ministry's ambitions using a combination of different discourses. In line with mental health legislation, and grounded in biomedical discourse, the strategy recognized the occasional need for coercion to manage risk or ensure necessary anti-psychotic treatment. The strategy also incorporated elements of relational social justice and some of the criticism from antipsychiatry, for instance when stating that “*inappropriate use of coercion can be traumatizing, worsen acute situations, destroy trust in the care system, and contribute to the patient not asking for help in the future.”* Recovery and psy discourse was alluded to when suggesting the use of coercion could be reduced by “*enabling persons with mental disorders to live a worthy life in their home community”* and “*by directing focus toward prevention and alternative voluntary solutions based on cooperation, on as equal a footing as possible, between users/family carers and health professionals*.” The strategy balanced its portrayal of the usefulness of antipsychotic medication by describing its efficacy as mixed: while useful for some patients, others experience debilitating side effects, and, if given involuntarily, medication “*may be experienced as very intrusive and constitute an additional mental burden”* (81). As such, the strategy was based on knowledge from biomedical, psy, recovery and experiential positions, which were all taken as valid, but for different patients and/or situations.

We found no sign of concrete plans for MFS following the launch of the strategy. In October 2012, the Joint Action wrote to the Minister about this continued lack of progress, stating that: “*we believe one reason why MFS is not prioritized and manifesting itself in the Hospital Trusts' plans is a lack of knowledge and experience [with MFS]”* (82). Thus, they pointed to inadequacies in Trusts' knowledge base as an explanation for why they failed to meet the Ministry's demands, without phrasing it as harsh criticism. Instead, they proposed to rectify this deficiency with proper user involvement, and suggested a dialogue conference where different stakeholders could develop solutions together, presumably based on the pooling of different sources of knowledge. In a positioning document of 2013, the Joint Action reiterated their position and added the argument of MFS as an improvement for those family carers who “*feel pressed to accept or pressurize [the patient] to take medication, despite their own concerns about medication and their wish to support the patient's preferences”* (88).

Several Hospital Trusts developed plans for reducing coercion in 2012–14 (84–87). As before, some of these discussed MFS, but none formulated actual plans for implementation and, also as before, this omission, which might be read as tacit resistance toward the Ministry's requirement, was not explained or justified.

#### A Bureaucratic Assertion of Authority

After instructing Health Authorities to implement MFS in 2010 and 2012, the Ministry made another attempt in 2015. At that point, the Commissioning Letter specified that MFS should be “*developed in close cooperation with user organizations”* and that the Trusts “*shall report plans for how [MFS] will be carried through by 1 March 2015”* (89).

As the only body to comply within the deadline, the Professional Advisory Council in the largest Regional Health Authority issued a plan (94). In it, they stated that antipsychotics “*should only be prescribed on clear indication and discontinued in absence of effect*” and that “*all patients shall, as far as is possible and responsible, be able to choose between treatment alternatives, including MFS.”* The plan thus paid attention to both psy and recovery orientations, but the caveat of “if possible and responsible” suggested that they wished to maintain the position of biomedically based professional authority. They also warned against establishing MFS as separate wards or units, which they described as “*a radical understanding of the assignment,”* and they stated it would be “*professionally irresponsible”* not to recommend or offer patients medication (94). In response, Mental Health Norway—the largest national mental health user organization —stated that separate MFS wards were indeed necessary, and described the Council as “*completely blind”* to patients' lack of real choice in current services. They added that “*the alternatives preferred by users are insufficiently researched to have an impact in the hierarchy of evidence,”* (95) thus criticizing this case of power imbalance between biomedical and experiential knowledge.

The Ministry followed up in November 2015 with an amended Commissioning Letter, phrased in clear, authoritative language: “*the Ministry finds it necessary to specify the assignment with deadlines”* for when MFS was to be realized (90), and specified that five units were to be in operation by June 2016. While MFS was described in terms of recovery in that it offered patients alternatives to medication that should include individualized plans for discontinuing medication in safe environments and at patients' requests, it was bureaucratic, authoritative discourse that dominated the tone of the letter. This time the Regional Health Authorities complied, and specified plans for MFS were developed and reported to the Ministry (91).

### The Public Debate

As shown in the previous section, discussions surrounding the development of MFS were largely among the Ministry of Health, Regional Health Authorities and Hospital Trusts, and user organizations. Opposition to their introduction was indirectly expressed through the lack of action on behalf of Health Authorities and Trusts. As MFS units were about to begin operation, however, a high-profile professor of psychiatry published an opinion piece in which he set out a range of arguments against MFS (96). This ignited a heated public debate that lasted for almost 2 years. His criticism, firmly founded on biomedical discourse, attracted support from a number of psychiatrists but was also countered by patients, psychiatrists, and other mental health professionals. Representatives from the Ministry did not take part in the debate. From the thematic analysis of the 75 texts, we identified five major themes, which were debated from different discursive positions, as described next.

#### The Health Minister's Decision vs. Evidence-Based Medicine

According to critics, the introduction of MFS failed to fully recognize scientific evidence that demonstrate that “*antipsychotics are useful for the great majority of patients with long-term psychosis”* (97) and that such medication can prolong patients' lives (98) and improve their symptoms, functioning, and quality of life. From the position of EBM (concerned with group level effects) it was suggested that MFS was “*a populist stunt from a the Minister concerned with showing how he “takes people seriously””* (99), that the Minister demonstrated “*a lack of respect for knowledge and research”* (100) and had “*let himself be manipulated to establish a service that does not follow the Directorate of Health's own guidelines [for psychosis treatment]”* (101). The Minister's decision was interpreted as, inadvertently or not, devaluing psychiatric expertise, as he would never instruct Trusts “*to establish chemotherapy-free care for cancer patients or medication-free heart treatment”* (102).

Such arguments were countered by MFS supporters, who, drawing on pharmaceutic-critical discourse, argued that the evidence for the efficacy of antipsychotics was nuanced, that they could have detrimental side effects, and that “*there is no doubt that pharmaceuticals kills many and that antipsychotics shorten lives a great deal”* (103). Some argued that it was important to see beyond a singular focus on EBM and, implicitly, biomedical discourse, and portrayed MFS as a useful tool to promote patient autonomy (104). Indeed, arguing with procedural justice, the Minister was commended for his “*clarification of current patient rights”* (105). MFS critics countered the emphasis on negative side effects of medication by stating that it could “*scare people in vulnerable situations and lead many to stop using medicines that are safe and effective”* (106). Showing great faith in EBM, one MFS critic was concerned that “*MFS isn't just a bad idea: it may fair and square end up introducing systematic malpractice. At worst, lives can be lost”* (101).

An editorial in the Journal of the Norwegian Medical Association suggested that the demand for MFS might partly stem from the hegemonic position of biomedical interventions combined with limitations in its scientific achievements concerning psychiatric diagnostics and treatment (107). This prompted response from MFS critics, defending psychiatry's biomedical achievements. One warned that an editor should be “*careful with making too categorical claims about the status of current knowledge,”* and that “*the knowledge about genetic, physiological and biochemical changes in severe mental disorders such as schizophrenia and bipolar disorder is fully on par with knowledge of many so-called somatic disorders”* (108). Another described the editor as “*uneducated*” and his position as “*ill-considered and principally questionable for an editor in a medical journal”* (109). Implicitly criticizing an antipsychiatry perspective, it was suggested that the editor had “*lost himself in the reflections of bygone philosophers*” instead of “*backing up the criticism of MFS”* (110).

#### The Ethics of Introducing MFS on the Basis of Current Evidence

The lack of biomedical evidence for non-medical treatment without the simultaneous use of medication was depicted as unethical by MFS critics. With a nod to experiential knowledge, it was argued that the only information about treatment completely without antipsychotics was “*how it was to suffer from psychosis before 1950, (a situation) to which we don't want to return”* (111). Consequently, it “*must be considered ethically dubious to take this option [medication] away from patients”* (97). While not addressing it directly, the need for coerced treatment to protect some patients was implied when MFS was described as posing a risk to services' ability to address a core problem of psychiatry: “*that the most severely ill patients often lack insight”* (101).

MFS supporters drew on biomedical discourse to counter this line of argument, referring to efficacy trials of antipsychotics showing that “*not everybody gets better with medication, and also a proportion of those who do not take any medication get better”* (112). “Open Dialogue” in Northern Finland was used as an example of treatment with minimal medication use yet with recovery rates around 80% (113).

#### MFS and the Most Vulnerable Patients

MFS critics argued that user organizations pushing for MFS focused on the rights of relatively well-functioning activists to the detriment of the most vulnerable patients. While MFS activists were able to speak out (102), those most vulnerable had “*limited ability to go to the barricades for guarantees of treatment in line with the best clinical standards”* (110). With those standards based in EBM, MFS critics thus portrayed themselves as the real protectors of the most severely ill, casting doubt on the relevance of activists' experiential knowledge. This elicited strong, personal responses. One MFS supporter presented her rejection of the biomedical approach as the very reason for her ability to advocate: “*I would have been one of them (physically and mentally damaged or even dead) if I hadn't, as a young patient, refused to follow the advice to take antipsychotics”* (114). Another expressed social justice discourse that the ability of vulnerable patients to have their voices heard was curtailed by services: “*When we protest, it is stated in our records that we are uncooperative and lack insight…When we argue matter-of-factly that [medication] has been tried with poor result, we get another diagnosis…People with psychotic experiences are indeed vulnerable, but we will not be told that we are incapable of standing on the barricades”* (115). Disagreement thus remained regarding which knowledge base should form basis for protecting the most vulnerable patients.

#### Appropriate Treatment With and Without Medication in Current Services

MFS critics acknowledged procedural social justice issues in form of the patient's rights to choose: “*there is no doubt that the patients themselves should decide what treatment to receive, including medication*” (109). It was noted that most patients with psychosis in fact choose to take antipsychotics (116). While MFS critics saw room for service improvement by “*stopping antipsychotics when they don't work*,” (117), this “*should take place in regular wards”* (116), and not “*as small “antipsychiatric islands” within each hospital trust”* (117). MFS supporters countered that choosing care without medication was not a real option in current services, due to coercive environments and treatment pressure (118). To the extent that they were able to choose, they argued, patients were usually left with three alternatives: “*to take medication voluntarily, to be medicated involuntarily, or to receive no care”* (119). A psychiatrist who endorsed the MFS initiative, described it as a potential “*correction”* to the current collusion between psychiatrists and the pharmaceutical industry, and to the exaggerated medication focus in current services which resulted in patients “*typically bringing with them a long shopping list of medicines… Many have been over-treated and mistreated”* (120).

The situation for those experiencing no or negative effects of antipsychotic medication had been central in both the Ministry's and the Joint Action's positions on MFS. MFS critics shared the concern for this group (111), estimating that around 20% of patients with schizophrenia were antipsychotic “non-responders.” They stated this in biomedical terms, suggesting that there are “*no markers today that can tell us who (non-responding) patients are before medication is tried*” (102), and that changing current medication practice “*is a risky idea for first episode patients”* (101). We found no specific discussion of how MFS critics related this to involuntary medication more generally. Coerced medication was central to MFS supporter's experiential arguments for why MFS was needed and why it should be delivered in separate wards. It was argued that the MFS critics' failure to take into account the lived experiences of coercive antipsychotic treatment “*makes a mockery of all those of us for whom medication is of no help, and who, on top of it all, have had our lives destroyed by being coercively inflicted with the medicines that you glorify*” (121).

#### “Medication-Free” Might Have Has Problematic Connotations

According to some of the critics, the label “medication free” had potentially damaging effects as it might “*implicitly signify that medication is dangerous and something to avoid. The introduction of MFS can create an attitude that by and large supports expressed skepticism toward treatment with medication”* (96). Here, pharmaceutic-critical discourse is read into the MFS label. Moreover, attaching this label to separate inpatient wards “*could increase an artificial divide between medication and a variety of psychosocial treatment forms”* (122), when in fact “*optimal treatment often includes both (approaches) and isn't an “either-or.” By starting MFS we send a powerful signal of the latter”* (122). Here MFS critics explicitly endorsed the coexistence of psy and biomedical approaches.

MFS supporters did not address this criticism. However, one of them suggested that while MFS would be beneficial to some patients, the ambition implied by the label “medication-free” could only be realized if it was also available for those treated involuntarily: “*MFS is so far not the answer for those of us living in an eternal, ubiquitous risk of being coercively “treated” behind closed doors.”* Still, she was confident that momentum was on her side, and directly addressing MFS critics she stated that “*you won't be able to stop this train that has—finally—left the station”* (121).

### The “Problem Representations” of the Three Main Stakeholder Groups

According to two first stages in the analysis, MFS in Norway evolved through a process where the Ministry of Health adopted core premises put forward by user organizations. Heavy opposition emerged once this new service was implemented through bureaucratic force. MFS was debated drawing on arguments from a range of discourses, but in the main, MFS critics framed their position in biomedical discourse, privileging EBM, while MFS supporters applied experiential and social justice discourse emphasizing coercive medication.

All this informed the third stage of analysis in which we distilled implicit problem representations (62) from the broader positions on MFS of the Joint Action and MFS supporters, the Ministry, and the MFS critics, in order to arrive and an understanding of why consensus seemed so unattainable. These results are illustrated in Table 2.

**Table 2:** Expressions of standpoints in the introduction of MFS.

	**The Health Ministry**	**Joint Action and MFS supporters**		**MFS critics**
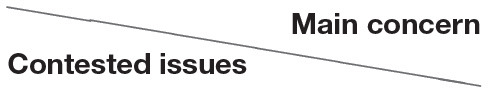	**Coercion rates**	**Patient autonomy**		**Evidence-based treatment**
Involuntary medication	Occasionally needed but should be reduced	Negative patient experiences; violates autonomy;		Not directly addressed; implicitly linked to insight
Non-response to antipsychotics	One reason for developing MFS	Central reason for making MFS mandatory		Can be addressed within current organization
Separate MFS wards	Instructed establishment of separate ward		Necessary to offer real treatment choice and avoid coercive environments	Creates unwanted divide between biological and psychosocial treatment
The ethics of MFS	MFS is needed to rectify negative patient experience		MFS imperative to improve patient autonomy	Treatment without scientific evidence is ethically dubious
Concern for the most severely ill patients	Implicit that they sometimes need interventions		Illustrate the failure of current care approaches. Need protection from coerced medication	Lack of insight can result in reluctance to receive evidence-based medication.
Implication of the MFS label	Not addressed		Enables reluctant patients to access treatment	Might create ‘antipsychiatric islands’ and stigmatize the use of medication
View of current care practice	Too high rates of coercion, can be rectified by various measures including MFS		Treatment without medication is unavailable, which curtails patient autonomy	Current services provides room for patient choice and treatment of non-responders
Evidence-base for MFS	Heterogeneous effects of medication and experiential evidence justifies MFS	Experiential evidence justifies MFS, as does the mixed scientific evidence for antipsychotics.		No relevant evidence to justify MFS but strong evidence for the efficacy of antipsychotics

The Joint Action's initial definition of the problem at stake was that some patients are coerced or pressurized to take medication that they do not wish to take or that does not benefit them, and that the effect of this was highly problematic on social justice grounds. This problem could be solved by developing MFS to give patients (including those in acute psychosis) the opportunity to freely accept or reject medication, and that rejection would not lead to “no care” being offered. An “estranged reading” of the Joint Actions' texts suggests that detail as to the practical handling of acute psychosis were insufficient, thus postponing some tricky feasibility and implementation issues.

The key problem inherent in the Ministry's position seemed to be the high compulsion rates, including for non-responders and those who experienced involuntary medication as a violation. This, they suggested, could be alleviated by Hospital Trusts developing MFS in cooperation with user organizations and mental health professionals, in ways that struck a balance between their varying concerns. Little detail was provided as to how this could be achieved, however, and we could not find a definition or description of “medication-free service” in any of the Ministry's texts.

MFS critics' expressed problem definition started with the solution offered by the other two groups: the implementation of MFS. They argued that, since MFS lacked the scientific evidence that antipsychotic medication has, it risked having negative consequences for patients, in particular for those lacking insight into their own illness. Implicitly, the solution to the problem as defined by this position seems to be to continue as before, along with incremental service improvement. An estranged reading of MFS critics' texts indicates that they omitted to set out a clear position on the issue of coercion, including experienced coercion, which, after all, was central to the problem definitions of both the Joint Action and the Ministry. Although emphasizing that non-response affected a proportion of patients, explicit exploration of connections between non-response, insight, and involuntary medication was lacking in MFS critics' texts.

## Discussion

The idea of providing MFS as an alternative to ordinary acute psychosis care was first suggested by a user organization. In 2010, the Ministry of Health instructed Health Authorities to implement such services as one of several measures to reduce coercion. The language in this first instruction lacked signal phrases from biomedical discourse and incorporated elements from social justice, psy, experiential and recovery discourse into the bureaucratic one. This can be interpreted as a sign that the Ministry accepted fundamental arguments of the user organizations, and there was no sign of there having been much consultation with the mental health professions prior to making MFS mandatory, which one might have expected when services were modified (123). This concurs with the criticism 6 years later that the Health Minister ignored biomedical evidence when requiring MFS.

When MFS did not develop, user and carer organizations formed a Joint Action that repeatedly reminded the Ministry of the failure to implement. It took 6 years—with increasingly firm and detailed instruction by the Ministry, drawing progressively more on their bureaucratic authority—before Health Authorities and Trusts complied and plans for MFS started to emerge. At that point, defenders of biomedical discourse aired their concerns in public media, sparking a heated debate as described above.

Two key aspects of this process have particular relevance beyond the Norwegian setting. First, the way in which experiential knowledge gradually entered the mainstream and how this prompted reactions from biomedical positions. Second, the discursive distance between stakeholder groups—as manifest in their key problem definitions—may, at least in part, be explained by the varying emphasis on experiential knowledge in general and experiences of involuntary medication for those without positive medication responses in particular.

### The Mainstreaming of Experiential Knowledge and the Biomedical Reaction

The Ministry's position represented an inclusion of patient perspectives into policymaking. This is not unique to the field of MFS and coercion, or indeed to mental health services. Across much of the world, patient and citizen involvement and consumerism have altered the roles of “experts” and shifted power positions, including those of government agencies (124). Crossley (22) describes how psychiatric patient accounts in the UK slowly grew in symbolic power, resulting in increased influence. This has manifested in user organizations joining forces also internationally, such as in their strategic negotiation that contributed to the removal of a “biomedical model” of health in the CRPD (8). In part, this was based on effective communication of personal experiences of mental health care (47), which facilitated the juxtaposition of “knowledge by experience” with other forms of knowledge. Similar processes were at play during the introduction of MFS in Norway, where experiential knowledge, supported by other discources, gained a firm foothold on the government's agenda for reducing coercion and for MFS.

We also observed changes in the discursive practices of user organizations. The experiential and procedural social justice discourse, and elements from antipsychiatry, dominating the early phase, often expressed as criticism of current care, were to some extent replaced by language that engaged more with psy, recovery and also biomedical discourse. This shift may have different explanations: it might have resulted from negotiated consensus within the broadly constituted Joint Action; represent a strategic change in rhetoric to secure continued support from the Ministry; or it might have been initiated to facilitate cooperation with health professionals when MFS seemed achievable. The Ministry incorporated social justice and experiential discourse promoted by user organizations, and even elements that may be read as representing antipsychiatry, into their bureaucratic writings. A range of knowledge bases and arguments were thus legitimized by the government, who expected health professionals and patient representative to collaborate on operationalizing local MFS.

We found no explanations in the texts as to why Health Authorities and Trusts for several years ignored the instructions to implement MFS. Local design of national policy is common in Norway, and given the Ministry's rather loose instructions, local implementation could have developed fairly autonomously. The fact that the Ministry had to repeat their instructions several times indicates reluctance to adopting MFS. This could reflect a “wait and see” approach in anticipation that the requirement would go away; the inability to envision safe arrangements for MFS during acute psychosis, or; tacit agreement with the views that were later expressed by MFS critics. This last element concurs with a recent qualitative study where Norwegian psychiatrists described MFS as unscientific and potentially undermining of medication regimes (14).

When MFS eventually was enforced, strong opposition was voiced. Critics publicly emphasized the superiority of EBM, portraying MFS as potentially unethical and criticizing the Minister for being “tricked” into letting other sources of knowledge influence policy. MFS critics claimed to be the true advocates of the most vulnerable patients. Given that modern psychiatric practice incorporates elements from a variety of treatment approaches and welcomes user involvement (125), the almost singular use of biomedical discourse was somewhat surprising. One interpretation is that in order to ensure good patient care, a reaction against the newly won position of non-medical discourse was deemed necessary. As such, it might represent a resistance or protest against a perceived hegemonic intervention (20) by the Ministry, whose support for MFS were altering the discursive order.

Again, this resembles processes elsewhere. The early conceptual work on the CRPD was, like MFS, developed in the relative absence of representatives for the traditional/hegemonic biomedical discourse (11) but, when published, it was met with considerable protest due to the implications for clinical practice (7, 12, 126, 127). Similarly, when involuntary medication was banned by the German federal court, loud protest followed from national psychiatric and nursing associations (128).

### Problem Representations in a Discursive Deadlock: How to Help Those Not Responding to Anti-psychotics

All three stakeholder groups recognized that non-response to medication represented a problem. As a shared concern, this could have facilitated dialogue and pragmatic agreements to bridge divergent perspectives and promote better care for this group of patients. This did not happen. Instead, the role of coercive medication for non-responders seemed to become a point at which discursive positions clashed and fronts were reinforced: MFS critics acknowledged non-response as an issue, but emphasized the biomedical superiority and did not explicitly discuss how this aligned with a concern for autonomy and social justice. MFS supporters, on the other hand, maintained that in a context of coercive practices, experiential knowledge was essential to understand what was at stake for this group of patients. This included the necessity of establishing MFS in separate wards, in order to support patients to make treatment decision without feeling pressurized. Such a position is, of course, highly critical of the psychiatric profession. Details as to how to provide safe care for this patient group during acute psychosis were not, however, addressed by any of the stakeholder groups.

The underlying problem definitions, based in different views on what constitutes legitimate knowledge, might therefore reflect a discursive distance that has been described by Jacob as the “*incommensurable worlds”* of “*patient experience and the psychiatric discourse”* (129). As such, MFS supporters and critics seem to be at a discursive deadlock, which was not helped by stakeholders omitting to address issues central to the positions of others. Unless ways to bridge these “worlds” can be found, there is reason to believe that this will continue to be an area of conflict in mental health care. If the Ministry's instruction to implement MFS was an attempt to break this deadlock by expecting service user organizations and mental health professionals to work together to implement MFS, it was not an immediate success, as our analysis shows. It would seem that for governments to successfully intervene or change the discursive order regarding the relative authority of different sources of knowledge in the mental health field, the role of coercion in the care for those not benefitting from medication is an area that needs considerable attention. Testing the effects of MFS in non-inferiority trials might also be a way forward that could be acceptable to all parties.

### Strengths and Limitations

Our analysis was limited to published texts related to the introduction of MFS in Norway: views expressed through other means are not included. While our document search was extensive, we cannot rule out that we may have missed relevant texts. The analysis provides insight into how central arguments and discourse were applied by different stakeholder groups. As such, our findings do not represent the views of individuals. All texts relate to processes in Norway, which might limit the applicability of findings to other settings. The data might be open to additional interpretations.

## Conclusion

Our analysis of texts related to the introduction of MFS in Norway shows that patients and user organizations influenced policy development through dynamic interplays between stakeholder groups. Elements of social justice and experiential discourse were incorporated into, and thereby protected by, bureaucratic discourse, and was integral to the Ministry's instruction to implement MFS. This challenged the discursive order, and was met with strong reactions, firmly based in biomedical discourse, that maintained the superiority of biomedical knowledge as the foundation for good patient care.

An irreconcilable discursive difference between the positions of MFS supporters and critics related to which source of knowledge should be authoritative when designing mental health services for acute psychosis care, and in particular for those for whom antipsychotic medication does not work as intended. If, as indicated by our findings, this constitutes a continuing area of conflict in mental health care, it follows that further testing of whether MFS' effectiveness in acute psychotic crisis is acceptable as compared to standard treatment, might go some way to resolve this discursive deadlock.

## Data Availability Statement

The raw data supporting the conclusions of this article are available in the public domain. The full list of sources will be made available by the authors, without undue reservation.

## Author Contributions

ON and JR jointly planned the study, contributed to analysis, interpretations and conclusions, and edited and approved the final manuscript. ON collected and analyzed documents and wrote the original manuscript. JR oversaw methodology and was responsible for funding acquisition and project administration.

## Conflict of Interest

The authors declare that the research was conducted in the absence of any commercial or financial relationships that could be construed as a potential conflict of interest.

## Footnotes

^1^Various terms used for the population group in voluntary and/or involuntary mental health care (such as patient, service user, consumer, survivor) usually reflect either a subsection of this group or the discourse within which a position is framed (3). We apply the term “patients” because this is the term used in the majority of texts analyzed. For the same reason, we apply the term “user organizations” to NGOs working for this group's interests.

## Abbreviations

MFS: medication-free inpatient services
CRPD: Convention on the Rights of Persons with Disabilities
EBM: evidence-based medicine.
